# Development of the gait outcomes assessment list for lower-limb differences (GOAL-LD) questionnaire: a child and parent reported outcome measure

**DOI:** 10.1186/s12955-021-01775-z

**Published:** 2021-05-05

**Authors:** Jennifer A. Dermott, F. Virginia Wright, Nancy M. Salbach, Unni G. Narayanan

**Affiliations:** 1 Division of Orthopaedic Surgery, Hospital for Sick Children, 555 University Avenue, Toronto, ON M5G 1X8 Canada; 2 Department of Physical Therapy, Faculty of Medicine, University of Toronto, Toronto, ON Canada; 3 Bloorview Research Institute, Holland Bloorview Kids Rehabilitation Hospital, Toronto, ON Canada; 4 Toronto Rehabilitation Institute, University Health Network, Toronto, ON Canada; 5 Child Health Evaluative Sciences, Research Institute, Hospital for Sick Children, Toronto, ON Canada; 6 Department of Surgery and Rehabilitation Sciences Institute, Faculty of Medicine, University of Toronto, Toronto, ON Canada

**Keywords:** Quality of life, Patient reported outcome measure (PROM), Orthopaedics, Paediatrics, Lower limb deficiency, Lower limb deformity, Limb length discrepancy

## Abstract

**Background:**

To develop a priority-based patient/parent reported outcome measure for children with lower-limb differences (LD) by adapting the Gait Outcomes Assessment List (GOAL) questionnaire.

**Methods:**

Guided by a conceptual framework of patient priorities, the GOAL questionnaire was iteratively modified and its sensibility evaluated by field-testing it on children with LD, and their parents. Cognitive interviews were conducted with a subgroup of these children, and an e-survey administered to a multidisciplinary group of health care professionals with expertise in paediatric LD. Findings were integrated to create the final version of the GOAL-LD.

**Results:**

Twenty-five children (9–18 years), 20 parents, and 31 healthcare professionals evaluated the content and sensibility of the GOAL, with an emphasis on the relevance and importance of the items to patients’ health related quality of life (HRQL). This resulted in the retention of 26 of the original 50 items, elimination of 12, modification of 12, and addition of seven new items. The new 45-item GOAL-LD questionnaire was shown to be sensible, and its content deemed important.

**Conclusions:**

The GOAL-LD questionnaire has a high level of face and content validity, and sensibility. It comprehensively captures the HRQL goals and outcomes that matter to children with LD and their parents. Following further psychometric evaluation, the GOAL-LD may serve as a much needed patient and parent reported outcome measure for this population.

**Supplementary Information:**

The online version contains supplementary material available at 10.1186/s12955-021-01775-z.

## Introduction

Pediatric lower-limb differences (LD) include a wide spectrum of congenital, developmental and acquired causes of limb deficiencies, deformities, and length discrepancies. Congenital lower-limb deficiencies, including the absence or shortening of a limb or part of a limb, have a reported incidence of 2–7 in 10,000 births [1]. About 1 in 1000 people have a clinically relevant length discrepancy greater than 2 cm [2]. Lower-limb deformities and length discrepancies are associated with a number of developmental conditions (e.g., Blount’s disease), or may be acquired as a consequence of partial or complete injuries to the growth plate secondary to fractures, infection or neoplasms. These conditions are associated with abnormal gait and increased biomechanical effort [3–5], altered appearance of the limb, and psychosocial consequences [3, 6]. Children with LD are faced with a variety of treatment options and often undergo multiple interventions throughout their childhood; yet we know very little about how this may impact their health-related quality of life (HRQL) [7]. The benefits of these interventions, let alone their comparative effectiveness, are poorly quantified because there are no validated outcome measures developed for this population.

**Fig. 1:**
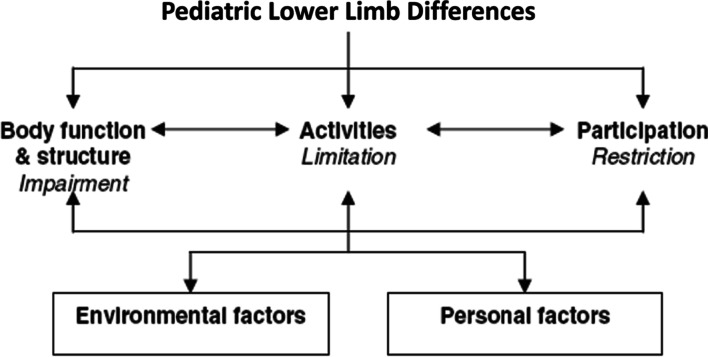
The international classification of functioning, disability and health (ICF) conceptualizes the consequences and outcomes of LD [8]

The International Classification of Functioning, Disability and Health (ICF) [8] is a useful framework to conceptualize the consequences and outcomes of LD, illustrated in Fig. 1. The management and evaluation of care should be informed by “multidimensional assessment leading to targeted interventions based on patient (parent) perceived needs” [9]. The ultimate goal of treating children with LD is to improve HRQL, optimizing function and maximizing participation, by addressing the physical, social and psychological effects of their LD. Research on pediatric LD has focused on the ICF domain of Body Functions and Structures [8] such as radiographic measures of limb alignment and length, post-operative complications, and time to heal [10]. Although these are important markers of the technical success of an intervention, one cannot assume that these correspond with HRQL outcomes that matter most to children/parents and are aligned with their priorities and goals. These are better captured in the ICF domains of Activity and Participation, using patient-reported outcome measures (PROMs) that are designed to measure these outcomes [11]. To date, generic measures of health status or HRQL (e.g., Child Health Questionnaire, [12]; PODCI [13]) have shown limited discriminative ability and responsiveness for this population, [6, 7, 14–16] highlighting the imperative for a more meaningful PROM for children with LD.

**Fig. 2:**
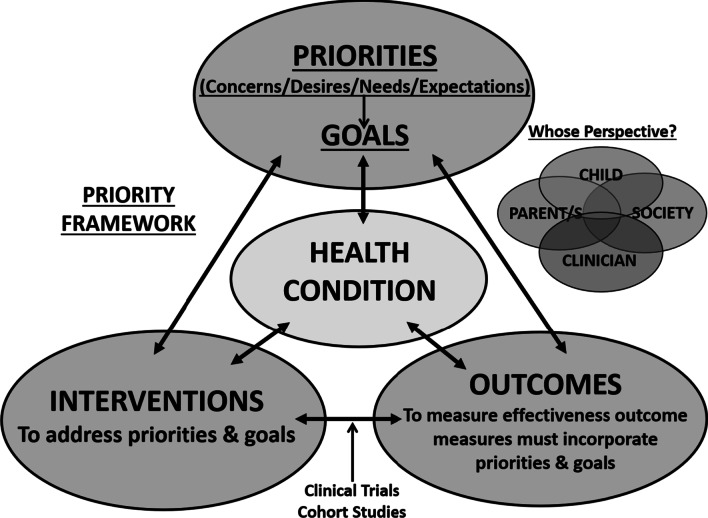
Priority framework for outcomes assessment [17, 18]: Adapted from https://lab.research.sickkids.ca/pscoreprogram/framework-of-patient-priorities/

The Priority Framework for Outcome Assessment [17, 18], depicted in Fig. 2, illustrates that to be meaningful to an individual, an outcome measure must incorporate their priorities and goals. To affect HRQL, interventions must address a patient’s goals, and effectiveness must be judged based on whether these goals were met. The Gait Outcomes Assessment List (GOAL) [19, 20] questionnaire is a multi-dimensional, self-administered child- and parent-report that was developed using the Priority Framework as its conceptual framework. The GOAL was created to evaluate outcomes based on the broad range of children’s and parents’ goals for gait related interventions for children with cerebral palsy, with a view to ultimately applying it to other childhood conditions associated with lower extremity impairments. The GOAL is a hybrid measure, combining the specificity of an individualized measure that identifies patient specific priorities or goals for treatment, with the standardization of a fixed item PROM. While individualized measures such as the Canadian Occupational Performance Measure (COPM) [21] and Goal Attainment Scaling (GAS) [22, 23] are well documented in pediatric rehabilitation outcomes research, most studies also employ a fixed item functional measure as a parallel tool [24] to evaluate intervention effectiveness at a group level and/or to provide predictive or discriminative information [25]. A questionnaire that combines the benefits of individualization *and* standardization is uniquely comprehensive and reduces the need to administer multiple questionnaires to patients.

The original version of the parent- and child-reported GOAL questionnaire [26] used as the starting point in this study, consists of 50 items across seven domains: (A) Activities of Daily Living (ADL) & Independence, (B) Gait Function & Mobility, (C) Pain/Discomfort/Fatigue, (D) Physical Activities, Sport & Recreation, (E) Gait Appearance, (F) Use of Braces & Assistive Devices, and (G) Body Image & Self-Esteem. Domains associated with tasks or activities use a 7-point ordinal scale anchored from 0: “extremely difficult/impossible” to 6: “no problem at all” with a 4-point modifier on how much assistance (from 0: “total” to 3: “independent”) is required to accomplish each task or activity. Respondents are asked to consider how they “usually perform” each item. Symptoms such as pain or fatigue are rated on a 6-point scale of frequency, from 0: “every day” to 5: “none of the time” as well as their intensity (0: “severe” to 2: “mild”). Domains that examine the respondent’s feelings use a 5-point ordinal scale from 0: “very unhappy” to 4: “very happy. Item scores are standardized (raw item score divided by total possible score for that item, multiplied by 100). Domain scores are the average of the standardized item score for each item in that domain, and the total score is the average of *all* the standardized item scores, reported from 0 to 100.

A key feature of the GOAL questionnaire is that for each item, the respondent also rates how important a goal it is to improve on that item using a 5–point scale from *not a goal* to *extremely important*. These importance ratings do not contribute to the total or domain score, but highlight, for each individual, which items are most important for improvement. Respondents may also specify additional goals and rate the importance of improving these.

Although the GOAL was developed and has been validated for children with ambulatory cerebral palsy [20], its focus on patient priorities and coverage of all domains of the ICF associated with gait-related problems [19] provides the foundation for developing a parallel measure for pediatric LD. Given that CP with its neurologic impairments is sufficiently different from LD, some of the content of the GOAL might not be as relevant to children with LD, and some important content to LD might be missing.

The aims of this study were to (1) evaluate the suitability of the items of the GOAL and its sensibility (face and content validity, comprehensibility, clarity of instruction, appropriateness of response scale, and ease of usage) from the perspective of children with LDs, their parents, and health care professionals (HCPs) with expertise in this population, and to (2) adapt the GOAL based on the input of these stakeholders to create the GOAL-LD. Permission to proceed with development of the GOAL-LD was granted by the GOAL’s developer (UG Narayanan, oral communication, September 2011).

## Methods

**Fig. 3:**
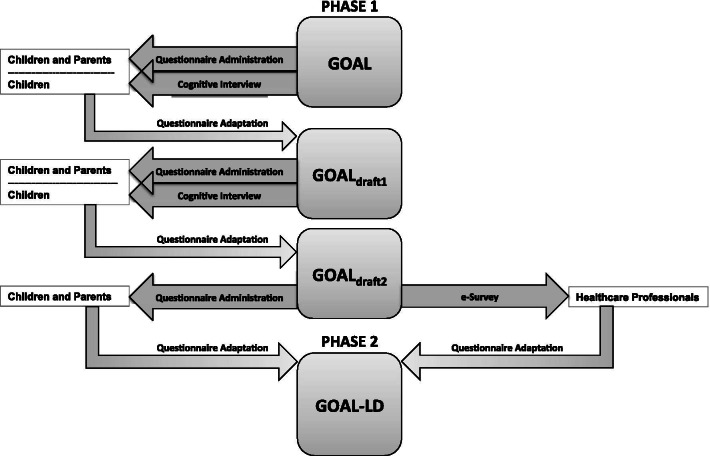
Flow diagram of study events

This two-phased study used an iterative process that considered all key stakeholders’ perspectives. Phase 1 involved patients and their parents, and Phase 2 involved health care professionals (HCPs) as is illustrated in Fig. 3.

### Participants

Children with LDs (ages 9 to 18 years) and/or their parents were recruited from the Limb Reconstruction Program at the Hospital for Sick Children and the Prosthetic Clinic of Holland Bloorview Kids Rehabilitation Hospital, both university-affiliated centres. Children were excluded if they had upper-limb involvement, neuromuscular conditions, or acute or systemic illness such sarcoma, or juvenile arthritis. The international multi-disciplinary group of content expert HCPs was identified through membership lists of the International Limb Lengthening and Reconstruction Society (LLRS), and the British Limb Reconstruction Society (BLRS), whose members represent a large number pediatric lower-limb reconstruction centres around the world. In accordance with the Research Ethics Board-approved protocol, children and parents provided written, informed consent to participate, while survey completion by HCPs implied consent.

### Phase 1: Children and parent perspectives

The GOAL questionnaire was administered by the first author to each of the participating children and/or their parents in a quiet room in the clinic setting. Participants scored each item and rated the importance of the goal to improve that item. The time to complete the questionnaire was documented. Children were invited to participate in a follow-up cognitive interview [27, 28]. These one-on-one interviews were guided by Feinstein’s framework of sensibility [29], which defines sensibility as “an aggregate of properties that make up the commonsense aspect of an instrument” [27]. It is increasingly being applied to evaluating the quality of questionnaires [30].

The first author conducted these semi-structured interviews based on the Cognitive Pretesting Model for Children [28] to probe whether the questionnaire resonated with them. The study-specific interview guide is included in Additional file 1. Also tested were the child’s understanding of words, phrases, and concepts that were deemed a priori by the development team to be potentially problematic (e.g., *What do you think “symmetry” means?)*. Children were asked questions regarding the scale format and response options (e.g., *Did you feel you were able to find your answer in the list of possible answers listed?),* any items that should be added or eliminated, and their overall impressions of the questionnaire, including its length. This information was used to guide decisions about the acceptance, modification or elimination of items in subsequent versions of the questionnaire (initial version = GOAL, second version = GOAL-LD_draft1_, and third version = GOAL-LD_draft2_)_,_ as described in the analysis. Recruitment was staged eight weeks apart so that each new version of the questionnaire was piloted with a different group of children and their parents.

### Phase 2: Health care provider (HCP) perspectives

HCPs received an introductory e-mail which included the GOAL-LD_draft2_ as an attachment and a link to an online feedback survey built for the study using FluidSurveys [31]. The e-survey asked HCPs to rate each of the proposed GOAL-LD questionnaire items as: *accept;* *accept with modification* (*and state the modification);* or *reject* *(and provide rationale for rejection*). This was an adaptation of an item reduction approach used successfully by one of the authors in development of a previous outcome measure [32].

Additionally, HCPs were asked to list other items that should be included, and were invited to respond to four open-ended questions: (1) *What do you consider to be the strengths of the GOAL-LD?;* (2) *What do you consider to be the weaknesses of the GOAL-LD?;* (3) *Would you consider use of the GOAL-LD in your clinical practice?;* and (4) *Would you consider using the GOAL-LD for research purposes?* The online survey platform recorded the geographic location (country) of respondents when their computers’ privacy settings permitted.

### Data analyses and iterative adaptation

Analysis of the completed questionnaires in combination with the children’s cognitive interview responses was conducted during Phase I after each stage of recruitment and informed the subsequent version of the questionnaire. Quantitative data analyses were performed using R for Mac OS X [33]. Children’s detailed responses from the cognitive interviews were collated into a single document, allowing general themes to be identified.

#### Phase 1

Descriptive statistics (mean, median, standard deviation [SD], range) were calculated separately for children’s and parents’ item scores and importance ratings for each iteration of the questionnaire. There are no published standard cut off thresholds to define “ceiling” or “floor effects”. Items were judged to perform poorly and considered for elimination if both child and parent item mean scores were within 0.5 of the maximum score (i.e., 5.5 to 6.0 on a 6-point scale), suggesting poor discriminative ability and unresponsiveness to change (ceiling effect). If an item does not have room for improvement, it is unlikely to be a goal for improvement. Similarly, items were considered for elimination if both the child and parent importance ratings were less than 0.5/4 on average. These extreme cut-off values were chosen to highlight the worst performing items, which were then examined alongside cognitive interview results to assess opportunities to modify, rather than prematurely eliminating items that may resonate with other children. Modified items and new item suggestions were considered in terms of redundancy and fit within the measurement concept and incorporated as appropriate into the next GOAL iteration to examine their performance.

The total number of child and parent participants and the number of GOAL-LD questionnaire iterations was based on achieving informational saturation, i.e., the point at which new suggestions related to content adaptation or to which novel input pertaining to sensibility were no longer forthcoming [34].

#### Phase 2

The HCP responses to the GOAL-LD_draft2_ were summarized for each item, and acceptance category percentages calculated. Items with at least 90% acceptance were retained in the GOAL-LD. Conversely, items with more than 10% *reject* responses were considered for elimination. The final decision about an item’s fate also considered the child/parent responses to the GOAL-LD_draft2_ to obtain further feedback on its performance. New items suggested were adopted based on whether they fit conceptually and were not redundant. Responses to the open -ended questions related to the questionnaire’s strengths/weaknesses and clinical/research utility were compiled into a single document, allowing general themes to be identified.

The child/parent responses to the GOAL-LD_draft2_ and the results of the HCP e-survey were considered together in the development of the final version of the GOAL-LD.

## Results

Forty-five participants, including 25 children (14 girls) with a mean age of 13.7 years (9.0 to 17.9 years) and 20 parents (16 mothers), were enrolled in Phase 1. Table 1 provides a summary of the participants’ age (children) and gender (parents and children), categorized by the questionnaire version they were administered. Sixteen children had a congenital LD (e.g., fibular hemimelia), 5 acquired (e.g., post-traumatic growth arrest), and 4 developmental (e.g., genu varum). Total leg length discrepancies ranged from zero to 100 mm, and deformity [35] ranged from none to greater than 15 degrees of malalignment. Additional file 2 further details children’s diagnostic characteristics. Of the 25 children enrolled, one opted to review but not complete the GOAL questionnaire and agreed to participate in the cognitive interview. In total, 13 children participated in the cognitive interviews (8 for the GOAL and 5 for the GOAL-LD_draft1_). Thirty-one HCPs (81% orthopedic surgeons; 52% North American) completed the Phase 2 survey that reviewed the GOAL-LD_draft2_. A demographic summary of HCP respondents is provided in Table 2.

**Table 1:** Phase 1 participants by questionnaire version administered

Questionnaire administered	Children				Parents
	GOAL completion (n)	Mean age (SD) Range	Cognitive Debrief^a^ (n)	Mean age (SD) Range	GOAL completion (n)^c^
GOAL	12 (7 girls/5 boys)	14.8 years (2.3) 12–17 years	8 (5 girls/3 boys)	15.7 years (2.6) 13–17	11 (9 mothers/2 fathers)
GOAL-LD_draft1_	5 (3 girls/2 boys)	11.8 years (2.6) 9–16 years	5 (2 girls/3 boys)	13.2 (2.7) 10–16	3 (2 mothers/1 father)
GOAL-LD_draft2_	7 (4 girls/3 boys)	13.4 years (2.4) 9–17 years	n/a	n/a	6 (5 mothers/1 father)
Total	24b (14 girls/10 boys)	13.7 years (2.6) 9–17 years	13 (7 girls/6 boys)	14.6 (2.5) 10–17	20 (16 mothers/4 fathers)

^a^Cognitive debrief sample is a subsample of the children who consented to participate

^b^Total study enrollment was 25 children. One child opted not to complete the questionnaire (GOAL-LD_draft1_) but did participate in a cognitive debrief interview

^c^Each parent completed a child-parent dyad

**Table 2:** Demographics of phase 2 HCP respondents (n = 31)

Demographics	n (out of 31)
*Healthcare profession*	
Pediatric orthopedic surgeon	25
Physical Therapist	4
Other: physical therapy practitioner, nurse practitioner	2
*Experience working with children with LDs (years)*	
< 5	6
5 to < 10	9
10- < 15	5
> 15	11
*Country*	
Canada	5
United States	11
United Kingdom	10
Europe	3
Unknown	2

HCP, health care professional; LD, lower limb difference

### GOAL content adaptation

#### Item addition

In total, seven new items were added, six derived from children’s and parents’ suggestions. These were retained in the final iteration based on the items’ subsequent scoring performance and the HCPs’ ratings. An item about *wearing a prosthesis* was introduced based on HCPs’ recommendations and was included to increase the questionnaire’s generalizability.

#### Item modification

Three items were modified to increase their level of difficulty (e.g., *carrying an object while walking (e.g., toy, doll, book, cellphone)* became *carrying heavy objects while walking (e.g., grocery bags, several schoolbooks).* Other examples of modifications through the iterations include: (1) three items that were split to improve their specificity (2) two items that were combined to minimize redundancy, and (3) 3 items that were moved to a different domain where the development team felt that they fit better conceptually. Six modifications were directly informed by HCP recommendations and involved wording to make items more explicit (e.g., *walking on slippery or icy surfaces* became *walking on wet, slippery or icy surfaces)* or to facilitate international utility (e.g., including metric and imperial measurements).

#### Item elimination

In total, 12 original GOAL items were eliminated of which six were in the ADL category and removed due to poor item performance in field-testing. *Walking for more than 15 min* was eliminated because in the cognitive interviews, children could not distinguish this item from *walking for more than 250 m.* The latter was retained because children were better able to conceptualize 250 m (“from swimming”, “from track and field”) and it had lower item scores (i.e., considered more difficult). One item was eliminated based on HCP feedback related to perceived redundancy, and the development team agreed that *moving quickly when in a hurry* overlapped with *running fast* since both items were speed related. The latter was retained based on an overall lower item score and wider distribution (range) of scores within the GOAL-LD_draft2_.

An overview of the content adaptations is provided with full item-by-item details by iteration, in Additional file 3.

### Sensibility evaluation

#### Comprehensibility

No participant asked for assistance or language clarification during GOAL or GOAL-LD_draft1_ administration. During cognitive interviews, all children demonstrated that they could read and understand the meaning of words, phrases, and concepts in the questionnaire.

#### Clarity of instruction

Five participants indicated they had not read the instructions prior to completing the questionnaire. Further, it became clear that instructions with respect to item importance ratings were problematic. The intended purpose of these ratings was to capture how important a goal it was to improve on the item, allowing identification of items that contribute most to treatment related decision-making. During the cognitive interviews, many respondents had a more generalized interpretation of importance, considering how it related to daily living overall rather than the importance as a goal for improvement. Written instructions were modified in the GOAL-LD_draft2_ to improve clarity.

#### Suitability of the response scale

In the original version of the questionnaire (GOAL), the recall period (in the past 4 weeks) was problematic for the domain of Physical Activities, Sport & Recreation, as some of these items were seasonal and many respondents checked the option “*I did not do this in the past 4 weeks”*. One child commented *“I’ve been living with this a long time, I know how these activities affect me even if I haven’t done them in the past 4 weeks”. *Thus, in the GOAL-LD_draft2_, the instructions were adapted and the respondents were instructed to consider the past year for the activities in this domain. No further changes were suggested related to the response scale.

#### Ease of usage

Nine children (69%) regarded the questionnaire as easy to complete. Three (23%) felt the questionnaire was too long. Time required for children to complete the questionnaire versions varied from 12 to 19 min.

Overall, 23 of 30 HCP respondents (77%) stated they would consider using the questionnaire in their clinical practice and 27 (90%) would apply it for research purposes. Twenty-five HCPs who identified strengths of the GOAL-LD_draft2_ based on its content, with 13 (52%) commenting that its comprehensiveness was an asset. However, 15 of 31 HCPs (48%) responded that the amount of time required to administer the GOAL was “too long”.

### The resulting GOAL-LD

The final iteration, the GOAL-LD, reflects the cumulative results of Phases 1 and 2. It contains 45 items organized into six domains and both the child and parent version are available in Additional file 4 and 5 respectively. Seven new items were added, 12 were eliminated, and 26 of the original 50 items were retained, as detailed in Additional file 3.

## Discussion

End users should be involved in the development of PROMs to ensure they are relevant and meaningful. The intent of this study was to create an LD-specific HRQL outcome measure using a systematic iterative process of review and adaptation that involved all key stakeholders. Administration of the original GOAL to children with LD and their parents revealed that some items, particularly in domain A) ADLs & Independence, did not pose a problem and consequently were not important goals for improvement, despite subsequent modifications to make some items more challenging. Consequently, this domain was eliminated to avoid a ceiling effect. Retention of such items would impede the questionnaire’s ability to discriminate between children and restrict its sensitivity to change following interventions. This issue might explain the poor performance of other generic function measures such as the CHQ [6] and Activity Scale for Kids (ASK) [16, 36] when used in this population as many of their items pertain to ADLs. For example, the ASK has several questions related to personal care, dressing, standing skills, and transfers, which the results of this study indicate are not generally affected by LDs.

The decision to adapt the GOAL for children with LDs, as opposed to creating an entirely new measure, was made for several reasons. The content of the original GOAL was generated, in part, from review of existing outcome measures that included measures previously used for children with LDs (i.e., ASK [36] PODCI [13]). Further, working from the GOAL as a template required a shorter time frame than creating a new measure. The use of cognitive interviews in our study, and the opportunity for children, their parents, and HCPs to provide suggestions for modifications and new items, ensured the adaptations through the various iterations of the GOAL-LD were well informed. Cognitive interviewing is a critical component of outcome measure development that strongly complements field-testing of a questionnaire [28]. Children in this study were keen to talk about their personal experiences related to their LD and six new item suggestions were generated in this manner (e.g., *wearing my choice of clothing, standing for a long time*).

Domains that consistently performed well and required minimal content adaptation were B) Pain/Discomfort/Fatigue and F) Body Image and Self-Esteem. Moreover, these items were rated as the most important goals across all versions of the GOAL. This makes sense because for many children and their parents, eliminating the visible deformity and its psychosocial consequences is their primary motivation for reconstructive surgery [7, 37].

Of the seven new items added, two relate to adaptations not typically used for children with CP (i.e., *use of a shoe-lift*, and *use of a prosthesis*). Concern that some of the language might be too difficult was reflected in HCPs’ comments but was not substantiated by children’s responses in cognitive interviews or during questionnaire completion. While half the HCPs believed that the length of the GOAL might present a challenge, most children felt it was appropriate. This highlights the importance of involving the intended respondents in the development of PROMS. The questionnaire is intended to be self-administered by children with LD and their parents and completed at home or in the out-patient waiting room, so that the time required to complete the questionnaire (range: 12–19 min) should not be difficult to accommodate, even in high-volume clinical settings.

The GOAL-LD was developed in children aged 9–18 years of age. For children under 9 years, one would expect the parent version to prevail, although some younger children might be able to self-report or complete the questionnaire with the help of a parent. This would need to be established in future validation work. The child version of the GOAL-LD captures the patients’ lived experience. The parent version captures the parent/s’ perspective of their child’s experience, including the child’s symptoms as observed by the parents or reported to them by the child. Considering the unique perspectives of children with LD and their parents reflects a family-centered care view in which both the patient and the parent each have the opportunity to collectively guide healthcare decision-making.

### Limitations

Although all key HCPs that work with children with LD were represented in our study (i.e., orthopaedic surgeons, physical therapists (PTs), and nurse specialists), our sample was comprised primarily of orthopaedic surgeons (81%). It is not surprising that surgeons are over-represented in this sample, since they are the key HCP in the current model of care to inform treatment, and most often serve as primary investigators in related research initiatives. Their participation in the development of this outcome measure is key to its future uptake. The PT perspective is well represented in the development of this outcome measure as 3 of the study team members are PTs.

The utility of a validated condition-specific outcome measure is limited to the condition for which it was designed. Although children with LDs are different from children with CP, pilot testing of the GOAL-CP on a sample of children with LDs provided sufficient justification to use the GOAL-CP content as an initial item pool, which was subjected to extensive iterative steps to ensure that the final GOAL-LD content was appropriately relevant and comprehensive for children with LD.

The cut off thresholds used to consider an item for elimination (5.5 to 6.0 on a 6-point scale for mean item scores or 0–0.5 on a 4-point scale for mean importance scores) were based on the assumption that these mean scores left no room for improvement or held no relative importance to respondents. Since there are no published cut off threshold criteria, these thresholds were not used in isolation to eliminate items, but rather to flag those items that were likely to demonstrate ceiling effects or were irrelevant. These items were assessed further during the cognitive interviews to explore whether modifying them would make them more useful, before a decision was made to eliminate them.

The GOAL-LD aims to capture the current status or performance on each item and uses a 4-week recall period. However, for the domain of Physical Activities, Sports and Recreation, respondents are asked to consider their performance in the “past year” as many of these activities may be seasonal. A longer recall period (within the past year) ensures that items in this domain are not left unanswered because of the lack of opportunity to perform the activity in the previous 4 weeks. Although the recall period does not impact the total score, we do want to make sure that the intent to capture current status is well understood by the respondents. If functional status has changed in the past year, the respondent should consider their most recent experiences. Future validation work will assess if the intent of the recall period needs to be more explicit in the instructions. The longer recall period might introduce errors due to recall bias. This will be explored by the assessment of test–retest reliability during further validation of the GOAL-LD.

The performance of the final HCP-guided adaptation (GOAL-LD) was not evaluated with children and parents. However, it is reassuring that content revisions between the third and final iteration were minimal. For example, *lineup* became *lineup/queue*, and *slopes* become *ramps/hills*.

## Conclusion

The GOAL-LD is a promising new outcome measure for comprehensively evaluating the physical and psychosocial wellbeing of children with LD, aged 9–18 years. This paper focuses on the development of the GOAL-LD, its face and content validity, and highlights its sensibility. The GOAL-LD is presently undergoing psychometric evaluation of its reliability, construct validity and responsiveness in an international, multi-center study (UG Narayanan, L Donnan, oral communication, 2016) of children with LD and their parents. If validated, the GOAL-LD will facilitate individualized goal-setting and shared decision making about the choice and timing of intervention, while also serving as a meaningful HRQL outcome measure for pediatric LD in clinical and research contexts.

## Supplementary information

**Additional file 1**. Cognitive interview guide

**Additional file 2**. Child participant demographics.

**Additional file 3**. Summary of content adaptation through each iteration of the GOAL (Phase 1 and 2 feedback).

**Additional file 4**. GOAL-LD Child version.

**Additional file 5**. GOAL-LD Parent version.
