# Supplementary material for: Development of the gait outcomes assessment list for lower-limb differences (GOAL-LD) questionnaire: a child and parent reported outcome measure
Source: Health Qual Life Outcomes. 2021 May 5;19:139. doi: 10.1186/s12955-021-01775-z (PMC8097808; doi:10.1186/s12955-021-01775-z)
Supplement: Supplementary file 1 — Additional file 1. Cognitive interview guide [file 12955_2021_1775_MOESM1_ESM.docx]

Development of the Gait Outcomes Assessment List for Lower-Limb Differences (GOAL-LD) Questionnaire: A Child and Parent Reported Outcome Measure

*Health and Quality of Life Outcomes*

Jennifer A. Dermott, Virginia Wright^*^, Nancy M. Salbach, Unni G. Narayanan^*^ (^*^co-senior authors)

Corresponding author: Jennifer A. Dermott, Hospital for Sick Children, 555 University Avenue, Toronto, ON, M5G 1X8 [jennifer.dermott@sickkids.ca](mailto:jennifer.dermott@sickkids.ca)

**Additional file 1**

**Cognitive interview guide**

Thank you for agreeing to complete the GOAL and to give me feedback on what you think of this questionnaire and how we can make it better. Your feedback will help make sure the GOAL asks questions that are meaningful and important to you and that the wording of the instructions, the questions, and the answer scale are very clear and easy to understand.

**Task 1: Questionnaire completion**

Please use the black pen to complete every item of the questionnaire. As you read each question, if there are any words or sentences that you do not completely understand, please highlight these words or sentences with the provided highlighter. If there is anything you would like me to explain, please ask. If there is anything you would like to share your thoughts on, for example something you really like or don’t like, let me know, either right away as you think of it, or circle it and we can talk about it at the end, when you are finished the questionnaire. If you have any other ideas that you think should be included in this questionnaire, please write these on the last page of the questionnaire.

**Task 2: Instructions**

Let’s look at the 4 instructions at the top of page 1.

- *Are these easy to understand?*
- *What does the word ‘mobility’ mean to you?­­­­­­­­­­­­­­­*

Let’s look at the instructions at the top of page 2.

- *In your own words can you explain what is being asked of you?*
- *What does the phrase “usually” mean to you?*

**Task 3: Scale**

Let’s take a look at the possible answers listed across the top of page 3.

There are 7 choices ranging from extremely difficult/impossible (0) to no problem at all (6).

- *Did you feel you were able to find your answer in the list of possible answers listed?*
- *Do you think there are too many options, not enough options, or is it just right?* (if they answer too many or not enough*, how would you change it?)*
- *How would you describe the difference between’ very difficult’ and ‘extremely difficult’?*

Now let’s look at the importance rating scale on this page. There are 5 choices ranging from ‘not a goal’ to ‘extremely important’.

- *Are there too many options, not enough options, or is it just right? (if too many or not enough, how would they change it)*

Let’s look at the scale on page 5. Notice that this now includes a new column, “I did not do this activity in the past 4 weeks”.

- *Do you think this is a good or bad option to include in the scale? Why?*

Now let’s look at page 7. This scale is asking about your feelings. You have 5 choices from very (0) unhappy to (4) very happy.

- *Are there too many options, not enough options, or is it just right?* (if too many or not enough, *how would you change it?)*
- *In your own words what does “neither happy nor unhappy” mean to you?*

**Task 4: Items**

**Section A:** Let’s take a look at page 2.

- *Can you read #7 aloud? (note words of difficulty)*
- *Can you ask the same question in your own words?*
- *The question lists “toy, doll, book, cellphone” as examples. Can you think of other examples that you might use?*

Look at the next question (#8).

- *What does the word “manually” mean to you?*

Take another look at all the questions in section A.

- *Are there any questions that you don’t think belong in this section?*
- *Are there any questions that you would get rid of completely from this questionnaire?*
- *Is there anything you would add to this section?*

**Section B**: Let’s look at page 3.

- *Can you read #11 aloud?* (note words of difficulty)
- *Do you feel you understand a distance of 250 m?*

Now Compare #11 to #14.

- *Explain to me the difference between these questions.*

Read the next question (#15).

- *Is there a different way to say this?*
- *Which way do you prefer?*

Take another look at all the questions in section B.

- *Are there any questions that you don’t think belong in this section?*
- *Are there any questions that you would get rid of completely from this questionnaire?*
- *Is there anything you would add to this section?*

**Section C**: Let’s look at page 4.

- *Explain to me the difference between pain and discomfort.*
- *Can you read #27 aloud? (note words of difficulty)*
- *Is there a different way to say this?*
- *Which way do you prefer?*

This section asks about pain/discomfort in 5 different parts of your body.

- *Is it easy for you to think about each of these body parts separately?*
- *Would organize or ask this group of questions any other way?*

Take another look at all the questions in section C.

- *Are there any questions that you don’t think belong in this section?*
- *Are there any questions that you would get rid of completely from this questionnaire?*
- *Is there anything you would add to this section?*

**Section D:** Let’s look at page 5.

- *Can you read #30 aloud for me?* (note words of difficulty)
- *What does the word ‘participating’ mean to you?*
- *What does the word ‘gliding’ mean to you?*
- *Is there another way to ask this question?*

Look at #33.

- *Is there another way to ask this question?*

Take another look at all the questions in section D

- *Are there any questions that you don’t think belong in this section?*
- *Are there any questions that you would get rid of completely from this questionnaire?*
- *Is there anything you would add to this section?*

**Section E:** Let’s look at page 6.

Look at #41.

- *How else could you ask this question?*

Look at the last line at the bottom of the page.

- *What does the word “aspect” mean?*
- *Is there a different word that you would use*?

Take another look at all the questions in section E.

- *Are there any questions that you don’t think belong in this section?*
- *Are there any questions that you would get rid of completely from this questionnaire?*
- *Is there anything you would add to this section?*

**Section F:** Let’s look at the top of page 7.

- *Are there any questions that you don’t think belong in this section?*
- *Are there any questions that you would get rid of completely from this questionnaire?*
- *Is there anything you would add to this section?*

**Section G:** Let’s look at the bottom of page 7.

- *Can you read #47 aloud for me? (note words of difficulty)*
- *What do you think “symmetry” means?*
- *Would you ask this question (#47) any differently?*
- *Can you read #48 aloud for me?*
- *How would you ask this question in your own words?*

Take another look at all the questions in section G.

- *Are there any questions that you don’t think belong in this section?*
- *Are there any questions that you would get rid of completely?*
- *Is there anything you would add to this section?*

**Task 5: General**

- *How do you feel about the amount of time it took you to complete the GOAL?*
- *How easy or difficult did you find the GOAL to answer?*

There are two purposes of the GOAL. One is to measure the impact of your leg condition on your daily life*…how well do you think the GOAL does this?*

And two is to highlight the areas you think are most important and would like to change*…*

- *How well do you think the GOAL does this?*
- *Do you think if your leg condition was corrected your answers would look very different?*
- *Is there anything else you can think of that would make this questionnaire better?*
