# Supplementary material for: Development of the gait outcomes assessment list for lower-limb differences (GOAL-LD) questionnaire: a child and parent reported outcome measure
Source: Health Qual Life Outcomes. 2021 May 5;19:139. doi: 10.1186/s12955-021-01775-z (PMC8097808; doi:10.1186/s12955-021-01775-z)
Supplement: Supplementary file 2 — Additional file 2. Child participant demographics. [file 12955_2021_1775_MOESM2_ESM.docx]

Development of the Gait Outcomes Assessment List for Lower-Limb Differences (GOAL-LD) Questionnaire: A Child and Parent Reported Outcome Measure

*Health and Quality of Life Outcomes*

Jennifer A. Dermott, Virginia Wright^*^, Nancy M. Salbach, Unni G. Narayanan^*^ (^*^co-senior authors)

Corresponding author: Jennifer A. Dermott, Hospital for Sick Children, 555 University Avenue, Toronto, ON, M5G 1X8 [jennifer.dermott@sickkids.ca](mailto:jennifer.dermott@sickkids.ca)

| Additional File 2 Child participant demographics | | | | | | | | |
| --- | --- | --- | --- | --- | --- | --- | --- | --- |
| Child | Age (years) | Gender | Etiology | Side | Total Leg Length Discrepancy (mm) | Lower Limb Alignment on AP radiograph  (Deformity Grade^a^) | Location of Current EFT  (if applicable) | Location of Previous EFT  (if applicable) |
| 1 | 16 | F | Tibial pseudarthrosis (C) | Left | 56 | Valgus (1) | Tibia |  |
| 2 | 14 | F | Septic hip (A) | Left | 31 | Valgus (1) | Tibia |  |
| 3 | 16 | M | Fibular hemimelia (C) | Left | 79 | Physiological range of variation | Tibia | Tibia  Femur |
| 4 | 12 | M | Fibular hemimelia (C) | Right | 50 | Physiological range of variation |  |  |
| 5 | 16 | F | Hemihypertrophy (C) | Right | 36 | Physiological range of variation |  |  |
| 6 | 17 | F | Fibular hemimelia (C) | Right | 15 | Valgus (2) |  | Tibia x 2  Femur |
| 7 | 17 | M | Tibia vara (D) | Bilateral | 0 | Varus (2) | Tibia (Bilateral) |  |
| 8 | 17 | M | Genu varum (D) | Right | 40 | Varus (3) |  |  |
| 9 | 13 | F | Osteosarcoma (A) | Right | 66 | Varus (2) | Tibia |  |
| 10 | 13 | F | Fibular hemimelia (C) | Left | 86 | Valgus (3) |  |  |
| 11 | 16 | F | Fibular hemimelia (C) | Right | 35 | Physiological range of variation |  |  |
| 12 | 10 | M | Congenital Femoral  Deficiency (C) | Right | 73 | Valgus (2) |  | Femur x 2 |
| 13 | 13 | M | Genu valgum (D) | Bilateral | 0 | Valgus (2) |  |  |
| 14 | 10 | F | Fibular hemimelia (C) | Right | 50 | Physiological range of variation |  |  |
| 15 | 10 | F | Post-traumatic growth arrest (A) | Left | 56 | Physiological range of variation |  |  |
| 16 | 15 | F | Congenital pseudoarthrosis (C) | Right | 70 | Physiological range of variation | Tibia |  |
| 17 | 16 | M | Post-traumatic growth arrest (A) | Left | 60 | Valgus (2) | Tibia |  |
| 18 | 9 | F | Tibial hemimelia (C) | Right | 57 | Physiological range of variation | Tibia |  |
| 19 | 13 | M | Fibular hemimelia (C) | Right | 40 | Valgus (2) | Tibia | Tibia |
| 20 | 13 | M | Perthes (D) | Left | 25 | Physiological range of variation |  |  |
| 21 | 14 | M | Osteomyelitis (A) | Right | 99 | Varus (2) | Femur |  |
| 22 | 13 | F | Congenital Femoral  Deficiency (C)^b^ |  |  |  |  |  |
| 23 | 9 | F | Congenital Femoral  Deficiency (C) | Right | 92 | Valgus (2) |  | Femur |
| 24 | 17 | M | Tibial hemimelia (C) | Left | 100 | Varus (3) |  | Tibia x 2 |
| 25 | 13 | F | Posterior medial bow of tibia (C) | Left | 50 | Physiological range of variation |  |  |

*AP* Anterposterior; *EFT* External fixator treatment; *C* Congenital; *D* Developmental; *A* Acquired

^a^Grade 1 deformity corresponds with approximately 5-10 degree malalignment, Grade 2 with 10-15 degree malalignment, and Grade 3 with greater than 15 degree malalignment; ^b^child had amputation
