# Supplementary material for: Development of the gait outcomes assessment list for lower-limb differences (GOAL-LD) questionnaire: a child and parent reported outcome measure
Source: Health Qual Life Outcomes. 2021 May 5;19:139. doi: 10.1186/s12955-021-01775-z (PMC8097808; doi:10.1186/s12955-021-01775-z)
Supplement: Supplementary file 3 — Additional file 3. Summary of content adaptation through each iteration of the GOAL (Phase 1 and 2 feedback). [file 12955_2021_1775_MOESM3_ESM.docx]

Development of the Gait Outcomes Assessment List for Lower-limb Differences (GOAL-LD) Questionnaire: A Child and Parent Reported Outcome Measure

*Health and Quality of Life Outcomes*

Jennifer A. Dermott, Virginia Wright^*^, Nancy M. Salbach, Unni G. Narayanan^* (*^co-senior authors)

Corresponding author: Jennifer A. Dermott, Hospital for Sick Children, 555 University Avenue, Toronto, ON, M5G 1X8 [jennifer.dermott@sickkids.ca](mailto:jennifer.dermott@sickkids.ca)

**Additional File 3**

Summary of content adaptation through each iteration of the GOAL (Phase 1 and 2 feedback)

| **GOAL** | **GOAL-LD_draft1_** | | **GOAL-LD_draft2_** | | | **GOAL-LD** | | |
| --- | --- | --- | --- | --- | --- | --- | --- | --- |
| **A) Activities of Daily Living & Independence** |  | |  | | |  | | |
| Getting in and out of bed | **Retained** | | **Eliminated** | | |  | | |
| Getting in and out of a chair or wheelchair | **Retained** | | **Eliminated** | | |  | | |
| Standing at a sink or counter | **Retained** | | **Eliminated** | | |  | | |
| Showering or bathing | **Retained** | | **Eliminated** | | |  | | |
| Using the toilet | **Eliminated** | |  | | |  | | |
| Getting dressed | **Modified:**Putting your pants on one leg at a time | | **Eliminated** | | |  | | |
| Carrying an object while walking (e.g., toy, doll, book, cell-phone | **Modified:**Carrying heavy objects while walking (e.g., grocery bags, several school books) | | **Retained** | | | **Modified**  Moved from A) to B) | | |
|  | **Modified:**Balancing objects while walking (e.g., a cup of hot chocolate, a tray of food) | | **Retained** | | | **Modified**  Moved from A) to B) | | |
| Opening a door manually | **Modified**: Opening a door | | **Retained** | | | **Eliminated** | | |
| Picking up an object off the floor | **Retained** | | **Retained** | | | **Eliminated** | | |
| Getting in and out of a vehicle (e.g., unmodified car, van, or bus) | **Modified:**Getting in and out of a vehicle (e.g., car, van or bus) | | **Retained** | | | **Eliminated** | | |
| **B) Gait Function & Mobility** |  | |  | |  | | |  |
| Walking for more than 250 m (around 2 blocks or 2 football fields) | **Retained** | | **Retained** | | | **Modified:**Walking for more than 250 m/ 820 feet (around 2 blocks or 2 football fields) | | |
| Getting around at school (indoors) | **Retained** | | **Retained** | | | **Modified:**Getting around in crowded spaces (school, concert, shopping centre) | | |
|  | **Added:** Getting around in crowded spaces (concert, special event, mall) | | **Retained** | | | See above | | |
| Getting around at home | **Eliminated** | |  | | |  | | |
|  | **Added:** Standing in long lineups | | **Retained** | | | **Modified:** Standing for a long time (lineups/ queues, parade, concert) | | |
| Walking for more than 15 minutes | **Retained** | | **Eliminated** | | |  | | |
| Walking faster than usual to keep up with others | **Modified:**Moving quickly if I am in a hurry (e.g., to catch a bus, late for school) | | **Modified:**Moving quickly when in a hurry (e.g., to catch a bus, late for an event) | | | **Eliminated** | | |
|  | **Modified:**Keeping up with my friends while walking outdoors | | **Modified:**Keeping up with my friends | | | **Modified:** Keeping up with my friends while walking outdoors | | |
| Stepping around or avoiding obstacles | **Retained** | | **Retained** | | | **Retained** | | |
| Going up and down stairs | **Retained** | | **Retained** | | | **Retained** | | |
| Going up and down slopes | **Retained** | | **Retained** | | | **Modified:** Going up and down ramps/ hills | | |
| Walking on uneven ground (rough, rocky, sandy) | **Retained** | | **Retained** | | | **Retained** | | |
| Walking on slippery or icy surfaces | **Retained** | | **Retained** | | | **Modified:** Walking on wet, slippery or icy surfaces | | |
| **C) Pain/Discomfort/Fatigue** |  | |  | |  | | |  |
| Pain or discomfort in the feet or ankles | **Retained** | | **Retained** | | | **Retained** | | |
| Pain or discomfort in the lower legs | **Eliminated** | |  | | | **Restored:** Pain or discomfort in the lower legs (shin or calf) | | |
| Pain or discomfort in the knees | **Retained** | | **Retained** | | | **Retained** | | |
| Pain or discomfort in the thighs or hips | **Retained** | | **Retained** | | | **Retained** | | |
| Pain or discomfort in the back | **Retained** | | **Retained** | | | **Retained** | | |
| Feeling tired while walking | **Retained** | | **Retained** | | | **Retained** | | |
| Feeling tired during any other physical activities that I usually enjoy (e.g., swimming, running, horseback riding, or other) | **Retained** | | **Retained** | | | **Modified:**Feeling easily tired during physical activities that I enjoy (swimming, running, or other sports) | | |
| **D) Physical Activities, Sport & Recreation** |  | | | | | | | |
| Using playground equipment (ladders, monkey bars, slides, etc.) | **Modified**: Climbing (e.g., ladder or stepstool) | | **Retained** | | | **Modified:**Climbing (e.g., ladder, playground equipment, climbing wall) | | |
| Running | **Retained** | | **Modified**: Running fast | | | **Retained** | | |
| Participating in gliding sports (e.g., skating, rollerblading, skiing, skate/snowboarding) | **Retained** | | **Retained** | | | **Retained** | | |
| Riding a bike or tricycle (with or without training wheels) | **Retained** | | **Retained** | | | **Retained** | | |
| Swimming | **Retained** | | **Retained** | | | **Retained** | | |
| Participating in sports that require running (e.g., soccer, baseball, football, track) | **Retained** | | **Retained** | | | **Retained** | | |
| Participating in sports that require jumping (e.g., basketball, volleyball) | **Retained** | | **Retained** | | | **Retained** | | |
| Participating in dance or martial arts (e.g., karate, judo, taekwondo) | **Retained** | | **Modified**: Participating in gymnastics, dance or martial arts (e.g., karate, judo, taekwondo) | | | **Modified:**Participating in sports that require balance (e.g., gymnastics, dance, martial arts) | | |
| **E) Gait Appearance** |  |  | |  | | |  | |
| Walking with my feet flat on the ground | **Retained** | | **Retained** | | | **Retained** | | |
| Walking taller or more upright (less crouched or bent at the knees) | **Retained** | | **Retained** | | | **Retained** | | |
| Walking with my feet pointing straight ahead | **Retained** | | **Retained** | | | **Retained** | | |
|  |  | | **Added:**Walking without a limp | | | **Retained** | | |
| Walking without dragging my feet | **Retained** | | **Eliminated** | | |  | | |
| Walking without tripping and falling | **Retained** | | **Retained** | | | **Retained** | | |
| Wearing my choice of footwear (e.g., shoes, boots, sandals) | **Modified:** Moved from E) to G) | | **Retained** | | | **Retained** | | |
| **F) Use of Braces and Assistive Devices** |  |  | |  | | |  | |
| Wearing braces or orthotics (e.g., AFO) | **Modified:**Wearing braces or orthotics (e.g., AFO) | | **Added:**A shoe lift | | | **Retained** | | |
|  |  |  | **Modified:**A brace (e.g., AFO) | | | **Retained** | | |
|  |  | |  | | | **Added:**A prosthesis | | |
| Using a walking aide (e.g., walker, stick, cane, crutches) | **Retained** | | **Retained** | | | **Retained** | | |
| Using a wheelchair | **Eliminated** | | **Restored** | | | **Retained** | | |
|  |  | | **Added**: Other assistive devices (e.g., built-up bicycle pedal or________) | | | **Retained** | | |
| **G) Body Image & Self-Esteem** |  |  | |  | | |  | |
| The shape and position of my legs | **Retained** | | **Retained** | | | **Retained** | | |
| The shape and position of my feet | **Retained** | | **Retained** | | | **Retained** | | |
| The symmetry of my legs (in length and size) | **Retained** | | **Retained** | | | **Retained** | | |
|  | **Added:** Wearing my choice of clothing (e.g., shorts, skirts, bathing suits) | | **Retained** | | | **Retained** | | |
| The appearance of how I get around compared with others | **Retained** | | **Retained** | | | **Retained** | | |
| The way others feel about how I get around | **Retained** | | **Retained** | | | **Retained** | | |
| How I am treated by others | **Retained** | | **Retained** | | | **Retained** | | |

*GOAL* Gait Outcomes Assessment List; *AFO* ankle foot orthosis
