# Supplementary material for: Development of the gait outcomes assessment list for lower-limb differences (GOAL-LD) questionnaire: a child and parent reported outcome measure
Source: Health Qual Life Outcomes. 2021 May 5;19:139. doi: 10.1186/s12955-021-01775-z (PMC8097808; doi:10.1186/s12955-021-01775-z)
Supplement: Supplementary file 4 — Additional file 4. GOAL-LD Child version. [file 12955_2021_1775_MOESM4_ESM.pdf]

Jennifer A. Dermott, Virginia Wright*, Nancy M. Salbach, Unni G. Narayanan* (*co-senior authors)
Corresponding author: Jennifer A. Dermott, Hospital for Sick Children, 555 University Avenue, Toronto, ON, M5G 1X8 jennifer.dermott@sickkids.ca

Additional file 4
GOAL-LD Child version

GOAL-LD
Gait Outcomes Assessment List
(for Lower-Limb Differences)
Child Version

1. We are trying to learn more about you and any goals you may have for treatment of your leg condition
2. Please read the instructions on each page carefully
3. Please answer all questions by circling the number and checking the box that fits best
4. You may choose to add more items that are important to you at the end of the questionnaire

For example:

A) Activities of Daily Living & Independence								LEVEL of ASSISTANCE				IMPORTANCE of GOAL				
Consider how you <u>usually</u> perform each of the following activities.<br>1) Rate how **easy or difficult** it was for you to perform each of these activities over the past 4 weeks; **AND**<br>2) Choose how much **assistance** you required to help you perform these activities; **AND**<br>3) Select **how important a goal** it is for you to improve in each of the following activities.																
	Impossible						***No problem at all***	**TOTAL**	**MODERATE**	**MINIMAL OR SUPERVISED**	**INDEPENDENT**	**Not a goal**	**Not very important**	**Fairly important**	**Very important**	**Extremely important**
**3. Balancing objects while walking (e.g., a cup of hot chocolate, a tray of food)**	0	1	2	3	(4)	5	6	0	1	2	(3)			✕		

In the above example, usual performance for **'balancing objects while walking'** is rated **4 out of 6*;*** requires **no assistance*;*** and improving this is a ***fairly important*** goal.

Your Name: ____

Date of Completion (dd/mm/yy): ______________________________

A) Gait Function & Mobility

Consider how you **usually** perform each of the following activities.

1) Rate how **easy or difficult** it was for you to perform each of these activities in the **past 4 weeks**; **AND**
2) Choose how much **assistance** you required to help you perform these activities; **AND**
3) Select **how important a goal** it is for you to **improve** in each of the following activities.

During the past 4 weeks:	*Impossible*						*No problem at all*	WALKING AID REQUIRED: WHEELCHAIR	WALKER	TWO CANES OR CRUTCHES	ONE CANE /CRUTCH/HAND SUPPORT/ RAILING OR WALL	INDEPENDENT	IMPORTANCE of GOAL: Not a goal	Not very important	Fairly important	Very important	Extremely important
**1. Carrying heavy objects (e.g., grocery bags, several school books) while walking**	0	1	2	3	4	5	6	0	1	2	3	4					
**2. Balancing objects (e.g., a cup of hot chocolate, a tray of food) while walking**	0	1	2	3	4	5	6	0	1	2	3	4					
**3. Walking for more than 250 meters/820 feet (around 2 blocks or 2 football fields)**	0	1	2	3	4	5	6	0	1	2	3	4					
**4. Getting around in crowded spaces (e.g., school, a concert or the shopping mall)**	0	1	2	3	4	5	6	0	1	2	3	4					
**5. Standing for a long time (e.g., lineups, queues)**	0	1	2	3	4	5	6	0	1	2	3	4					

A) Gait Function & Mobility- continued								WALKING AID REQUIRED					IMPORTANCE of GOAL				
Consider how you **usually** perform each of the following activities.<br>1) Rate how **easy or difficult** it was for you to perform each of these activities in the **past 4 weeks**; **AND**<br>2) Choose how much **assistance** you required to help you perform these activities; **AND**<br>3) Select **how important a goal** it is for you to **improve** in each of the following activities																	
**During the past 4 weeks:**	*Impossible*						*No problem at all*	**WHEELCHAIR**	**WALKER**	**TWO CANES OR CRUTCHES**	**ONE CANE /CRUTCH/HAND SUPPORT/ RAILING OR WALL**	**INDEPENDENT**	**Not a goal**	**Not very important**	**Fairly important**	**Very important**	**Extremely important**
**6. Keeping up with friends while walking outdoors**	0	1	2	3	4	5	6	0	1	2	3	4					
**7. Stepping around or avoiding obstacles**	0	1	2	3	4	5	6	0	1	2	3	4					
**8. Going up and down stairs**	0	1	2	3	4	5	6	0	1	2	3	4					
**9. Going up and down ramps**	0	1	2	3	4	5	6	0	1	2	3	4					
**10. Walking on uneven ground (rough, rocky, sandy)**	0	1	2	3	4	5	6	0	1	2	3	4					
**11. Walking on wet, slippery or icy surfaces**	0	1	2	3	4	5	6	0	1	2	3	4					

B) Pain / Discomfort / Fatigue							INTENSITY				IMPORTANCE of GOAL				
Consider each of the following items.<br>1) Rate how **often** you experienced pain or discomfort or tiredness in the **past 4 weeks**; **AND**<br>2) Choose how **severe** the pain or discomfort was; **AND**<br>3) Select **how important a goal** it is for you to **reduce pain or discomfort or tiredness** in each of the following															
**During the past 4 weeks:**	*Every Day*	*Nearly Every Day*	*2 to 3 Times a week*	*Once a Week*	*Occasionally*	*None of the Time*	**SEVERE**	**MODERATE**	**MILD**	**NONE**	**Not a goal**	**Not very important**	**Fairly important**	**Very important**	**Extremely important**
**12. Pain or discomfort in the feet or ankles**	0	1	2	3	4	5	0	1	2	3					
**13. Pain or discomfort in the lower legs (shin, calf)**	0	1	2	3	4	5	0	1	2	3					
**14. Pain or discomfort in the knees**	0	1	2	3	4	5	0	1	2	3					
**15. Pain or discomfort in the thighs or hips**	0	1	2	3	4	5	0	1	2	3					
**16. Pain or discomfort in the back**	0	1	2	3	4	5	0	1	2	3					
**17. Feeling easily tired during physical activities that I enjoy (e.g., swimming, running, or other sports)**	0	1	2	3	4	5	0	1	2	3					
**Other pain:** ________________	0	1	2	3	4	5	0	1	2	3					

C) Physical Activities, Games & Recreation

Consider how you **usually** perform each of the following activities.

1) Rate how **easy or difficult** it was for you to perform each of these activities in the **past year**; **AND**
2) Select **how important a goal** it is for you to **improve** in each of the following activities

**During the past year:**	*Impossible*						*No problem at all*	**I have never tried this activity**	**IMPORTANCE of GOAL:** Not a goal	Not very important	Fairly important	Very important	Extremely important
**18. Running fast**	0	1	2	3	4	5	6	☐					
**19. Participating in gliding sports (e.g., skating, rollerblading, skiing, skate/snowboarding)**	0	1	2	3	4	5	6	☐					
**20. Riding a bicycle**	0	1	2	3	4	5	6	☐					
**21. Swimming**	0	1	2	3	4	5	6	☐					
**22. Participating in sports that require running (e.g., soccer, baseball, football, track)**	0	1	2	3	4	5	6	☐					
**23. Participating in sports that require jumping (e.g., basketball, volleyball, trampoline)**	0	1	2	3	4	5	6	☐					
**24. Participating in sports that require balance (gymnastics, dance or martial arts)**	0	1	2	3	4	5	6	☐					
**25. Climbing (ladder, playground equipment, wall climbing)**	0	1	2	3	4	5	6	☐					
**Other recreational or sporting activity: ___________________**	0	1	2	3	4	5	6	☐					

D) Gait Appearance

Consider each of the following items.

1) Rate how **much of a problem** you experienced with each of the following in the **past 4 weeks**; **AND**
2) Select **how important a goal** it is for you to improve in each

**During the past 4 weeks:**	*Impossible*						*No problem at all*	IMPORTANCE of GOAL: Not a goal	Not very important	Fairly important	Very important	Extremely important
**26. Walking with my feet flat on the ground**	0	1	2	3	4	5	6					
**27. Walking taller or more upright (less crouched or bent at the knees)**	0	1	2	3	4	5	6					
**28. Walking with my feet pointing straight ahead**	0	1	2	3	4	5	6					
**29. Walking without a limp**	0	1	2	3	4	5	6					
**30. Walking without tripping and falling**	0	1	2	3	4	5	6					
**Other aspect of my walking:** ________________	0	1	2	3	4	5	6					

E) Use of Braces and Assistive Devices

IMPORTANCE of GOAL

Consider each of the following items.

1) Rate how **you felt** about using each of the following in the **past 4 weeks**; **AND**
2) Select **how important a goal** it is for you to reduce or eliminate use of these devices

**During the** **past 4 weeks**:	***Very Unhappy***				***Very Happy***	**Not a goal**	**Not very important**	**Fairly important**	**Very important**	**Extremely important**		
**31. A shoe lift**	0	1	2	3	4						☐<br>☐	I have not been prescribed a shoe lift<br>I choose not to wear my shoe lift
**32. A brace (e.g., AFO)**	0	1	2	3	4						☐<br>☐	I have not been prescribed any type of brace<br>I choose not to wear my brace
**33. A prosthesis (artificial leg)**											☐<br>☐	I have not been prescribed a prosthesis<br>I choose not to wear my prosthesis
**34. A walking aide (e.g., walker, stick, cane, crutches)**	0	1	2	3	4						☐	I do not use any walking aides
**35. A wheelchair**	0	1	2	3	4						☐	I do not use a wheelchair
**36. Other assistive devices (e.g., built-up bicycle pedal or ________________)**	0	1	2	3	4						☐	I do not use any other assistive devices

F) Body Image & Self-Esteem						IMPORTANCE of GOAL				
Consider each of the following items.<br>1) Rate how **you felt** about each of the following in the **past 4 weeks**; **AND**<br>2) Select **how important a goal** it is for you to improve in each of the following						**Not a goal**	**Not very important**	**Fairly important**	**Very important**	**Extremely important**
**During the past 4 weeks:**	***Very Unhappy***				***Very Happy***					
**37. The shape and position of my legs**	0	1	2	3	4					
**38. The shape and position of my feet**	0	1	2	3	4					
**39. The symmetry of my legs (in length and size)**	0	1	2	3	4					
**40. Wearing my choice of footwear (e.g., shoes, boots, sandals)**	0	1	2	3	4					
**41. Wearing my choice of clothing (e.g., shorts, skirts, bathing suits)**	0	1	2	3	4					
**42. The appearance of how I get around compared with others**	0	1	2	3	4					
**43. The way others feel about how I get around**	0	1	2	3	4					
**44. How I am treated by others**	0	1	2	3	4					

Other Goals	IMPORTANCE of GOAL				
If there are any other goals (long or short term) that we have missed, please list them below **AND** select **how important a goal** it is for you to **improve** in each.					
Other Goals:	Not a goal	Not very important	Fairly important	Very important	Extremely important
**1.**					
**2.**					
**3.**					
**4.**					
**5.**					

Comments & Suggestions

Thank You
