# Supplementary material for: Development of the gait outcomes assessment list for lower-limb differences (GOAL-LD) questionnaire: a child and parent reported outcome measure
Source: Health Qual Life Outcomes. 2021 May 5;19:139. doi: 10.1186/s12955-021-01775-z (PMC8097808; doi:10.1186/s12955-021-01775-z)
Supplement: Supplementary file 5 — Additional file 5. GOAL-LD Parent version. [file 12955_2021_1775_MOESM5_ESM.pdf]

Development of the Gait Outcomes Assessment List for Lower-Limb Differences (GOAL-LD) Questionnaire: A Child and Parent Reported Outcome Measure
Health and Quality of Life Outcomes
Jennifer A. Dermott, Virginia Wright*, Nancy M. Salbach, Unni G. Narayanan* (*co-senior authors)
Corresponding author: Jennifer A. Dermott, Hospital for Sick Children, 555 University Avenue, Toronto, ON, M5G 1X8 jennifer.dermott@sickkids.ca

Additional file 5
GOAL-LD Parent version

GOAL-LD
Gait Outcomes Assessment List
(for Lower-Limb Differences)
Parent Version

1. We are trying to learn more about your child and any goals you may have for treatment of their leg condition
2. Please read the instructions on each page carefully
3. Please answer all questions by circling the number and checking the box that fits best
4. You may choose to add more items that are important to you at the end of the questionnaire

For example:

A) Activities of Daily Living & Independence

Consider how your child **usually** performs each of the following activities.
1) Rate how **easy or difficult** it was for your child to perform each of these activities over the past 4 weeks; **AND**
2) Choose how much **assistance** your child required to help them perform these activities; **AND**
3) Select **how important a goal** it is for you to have your child improve in each of the following activities.

	Impossible						No problem at all	LEVEL of ASSISTANCE: TOTAL	MODERATE	MINIMAL OR SUPERVISED	INDEPENDENT	IMPORTANCE of GOAL: Not a goal	Not very important	Fairly important	Very important	Extremely important
**3. Balancing objects while walking (e.g., a cup of hot chocolate, a tray of food)**	0	1	2	3	(4)	5	6	0	1	2	(3)			✕		

In the above example, usual performance for **'balancing objects while walking'** is rated **4 out of 6*;*** requires **no assistance*;*** and improving this is a ***fairly important*** goal.

Your Name: ______________________________
Relationship to the Patient:______________________________
Date of Completion (dd/mm/yy): ______________________________

A) Gait Function & Mobility

Consider how your child **usually** performs each of the following activities.

1) Rate how **easy or difficult** it is for your child to perform each of these activities in the **past 4 weeks**; **AND**
2) Choose how much **assistance** your child required to help them perform these activities; **AND**
3) Select **how important a goal** it is for you to have your child **improve** in each of the following activities.

**During the past 4 weeks:**	*Impossible*						*No problem at all*	WALKING AID REQUIRED: WHEELCHAIR	WALKER	TWO CANES OR CRUTCHES	ONE CANE /CRUTCH/HAND SUPPORT/ RAILING OR WALL	INDEPENDENT	IMPORTANCE of GOAL: Not a goal	Not very important	Fairly important	Very important	Extremely important
**1. Carrying heavy objects (e.g., grocery bags, several school books) while walking**	0	1	2	3	4	5	6	0	1	2	3	4					
**2. Balancing objects (e.g., a cup of hot chocolate, a tray of food) while walking**	0	1	2	3	4	5	6	0	1	2	3	4					
**3. Walking for more than 250 meters/820 feet (around 2 blocks or 2 football fields)**	0	1	2	3	4	5	6	0	1	2	3	4					
**4. Getting around in crowded spaces (e.g., school, a concert or the shopping mall)**	0	1	2	3	4	5	6	0	1	2	3	4					
**5. Standing for a long time (e.g., lineups, queues)**	0	1	2	3	4	5	6	0	1	2	3	4					

A) Gait Function & Mobility- continued

Consider how your child **usually** performs each of the following activities.

1) Rate how **easy or difficult** it was for your child to perform each of these activities in the **past 4 weeks**; **AND**
2) Choose how much **assistance** your child required to help them perform these activities; **AND**
3) Select **how important a goal** it is for you to have your child **improve** in each of the following activities

**During the past 4 weeks:**	*Impossible*						*No problem at all*	WALKING AID REQUIRED: WHEELCHAIR	WALKER	TWO CANES OR CRUTCHES	ONE CANE /CRUTCH/HAND SUPPORT/ RAILING OR WALL	INDEPENDENT	IMPORTANCE of GOAL: Not a goal	Not very important	Fairly important	Very important	Extremely important
**6. Keeping up with their friends while walking outdoors**	0	1	2	3	4	5	6	0	1	2	3	4					
**7. Stepping around or avoiding obstacles**	0	1	2	3	4	5	6	0	1	2	3	4					
**8. Going up and down stairs**	0	1	2	3	4	5	6	0	1	2	3	4					
**9. Going up and down ramps**	0	1	2	3	4	5	6	0	1	2	3	4					
**10. Walking on uneven ground (rough, rocky, sandy)**	0	1	2	3	4	5	6	0	1	2	3	4					
**11. Walking on wet, slippery or icy surfaces**	0	1	2	3	4	5	6	0	1	2	3	4					

B) Pain / Discomfort / Fatigue							INTENSITY				IMPORTANCE of GOAL				
Consider each of the following items.<br>1) Rate how **often** your child experienced pain or discomfort or tiredness in the **past 4 weeks**; **AND**<br>2) Choose how **severe** the pain or discomfort was; **AND**<br>3) Select **how important a goal** it is for you to have your child **reduce pain or discomfort or tiredness** in each of the following															
**During the past 4 weeks:**	***Every Day***	***Nearly Every Day***	***2 to 3 Times a week***	***Once a Week***	***Occasionally***	***None of the Time***	**SEVERE**	**MODERATE**	**MILD**	**NONE**	**Not a goal**	**Not very important**	**Fairly important**	**Very important**	**Extremely important**
**12. Pain or discomfort in the feet or ankles**	0	1	2	3	4	5	0	1	2	3					
**13. Pain or discomfort in the lower legs (shin, calf)**	0	1	2	3	4	5	0	1	2	3					
**14. Pain or discomfort in the knees**	0	1	2	3	4	5	0	1	2	3					
**15. Pain or discomfort in the thighs or hips**	0	1	2	3	4	5	0	1	2	3					
**16. Pain or discomfort in the back**	0	1	2	3	4	5	0	1	2	3					
**17. Feeling easily tired during physical activities that my child enjoys (e.g., swimming, running, or other sports)**	0	1	2	3	4	5	0	1	2	3					
**Other pain: __________________**	0	1	2	3	4	5	0	1	2	3					

C) Physical Activities, Games & Recreation

Consider how your child **usually** performs each of the following activities.

1) Rate how **easy or difficult** it was for your child to perform each of these activities in the **past year**; **AND**
2) Select **how important a goal** it is for you to have your child **improve** in each of the following activities

**During the past year:**	*Impossible*						*No problem at all*	**My child has never tried this activity**	Not a goal	Not very important	Fairly important	Very important	Extremely important
**18. Running fast**	0	1	2	3	4	5	6	☐					
**19. Participating in gliding sports (e.g., skating, rollerblading, skiing, skate/snowboarding)**	0	1	2	3	4	5	6	☐					
**20. Riding a bicycle**	0	1	2	3	4	5	6	☐					
**21. Swimming**	0	1	2	3	4	5	6	☐					
**22. Participating in sports that require running (e.g., soccer, baseball, football, track)**	0	1	2	3	4	5	6	☐					
**23. Participating in sports that require jumping (e.g., basketball, volleyball, trampoline)**	0	1	2	3	4	5	6	☐					
**24. Participating in sports that require balance (gymnastics, dance or martial arts)**	0	1	2	3	4	5	6	☐					
**25. Climbing (ladder, playground equipment, wall climbing)**	0	1	2	3	4	5	6	☐					
**Other recreational or sporting activity: ___________________**	0	1	2	3	4	5	6	☐					

D) Gait Appearance								IMPORTANCE of GOAL				
Consider each of the following items.<br>1) Rate how **much of a problem** your child experienced with each of the following in the **past 4 weeks**; **AND**<br>2) Select **how important a goal** it is for you to have your child improve in each												
**During the past 4 weeks:**	***Impossible***						***No problem at all***	**Not a goal**	**Not very important**	**Fairly important**	**Very important**	**Extremely important**
**26. Walking with his/her feet flat on the ground**	0	1	2	3	4	5	6					
**27. Walking taller or more upright (less crouched or bent at the knees)**	0	1	2	3	4	5	6					
**28. Walking with his/her feet pointing straight ahead**	0	1	2	3	4	5	6					
**29. Walking without a limp**	0	1	2	3	4	5	6					
**30. Walking without tripping and falling**	0	1	2	3	4	5	6					
**Other aspect of my child's walking: ________________**	0	1	2	3	4	5	6					

E) Use of Braces and Assistive Devices

Consider each of the following items.

1) Rate how you **feel** about your child using each of the following; **AND**

2) Select **how important a goal** it is for you to have your child reduce or eliminate use of these devices

**During the <u>past 4 weeks</u>:**	***Very Unhappy***				***Very Happy***	**IMPORTANCE of GOAL**<br>**Not a goal**	**Not very important**	**Fairly important**	**Very important**	**Extremely important**		
**31. A shoe lift**	0	1	2	3	4						☐<br>☐	My child has not been prescribed a shoe lift<br>My child chooses not to wear their shoe lift
**32. A brace (e.g., AFO)**	0	1	2	3	4						☐<br>☐	My child has not been prescribed any type of brace<br>My child chooses not to wear their brace
**33. A prosthesis (artificial leg)**											☐<br>☐	My child has not been prescribed a prosthesis<br>My child chooses not to wear their prosthesis
**34. A walking aide (e.g., walker, stick, cane, crutches)**	0	1	2	3	4						☐	My child does not use any walking aides
**35. A wheelchair**	0	1	2	3	4						☐	My child does not use a wheelchair
**36. Other assistive devices (e.g., built-up bicycle pedal) ________________)**	0	1	2	3	4						☐	My child does not use any other assistive devices

F) Body Image & Self-Esteem

						IMPORTANCE of GOAL				
Consider each of the following items.<br>1) Rate how you **feel** about your child using each of the following; **AND**<br>2) Select **how important a goal** it is for you to have your child reduce or eliminate use of these devices										
**During the <u>past 4 weeks</u>:**	***Very Unhappy***				***Very Happy***	**Not a goal**	**Not very important**	**Fairly important**	**Very important**	**Extremely important**
**37. The shape and position of my child's legs**	0	1	2	3	4					
**38. The shape and position of my child's feet**	0	1	2	3	4					
**39. The symmetry of my child's legs (in length and size)**	0	1	2	3	4					
**40. Wearing his/her choice of footwear (e.g., shoes, boots, sandals)**	0	1	2	3	4					
**41. Wearing his/her choice of clothing (e.g., shorts, skirts, bathing suits)**	0	1	2	3	4					
**42. The appearance of how my child gets around compared with others**	0	1	2	3	4					
**43. The way others feel about how my child gets around**	0	1	2	3	4					
**44. How my child is treated by others**	0	1	2	3	4					

Other Goals

If there are any other goals (long or short term) that we have missed, please list them below **AND** select **how important a goal** it is to have your child **improve** in each.

Other Goals:	IMPORTANCE of GOAL: Not a goal	Not very important	Fairly important	Very important	Extremely important
**1.**					
**2.**					
**3.**					
**4.**					
**5.**					

Comments & Suggestions

Thank You
